# Austrian treatment algorithms in HER2-positive metastatic breast cancer: a 2022 update

**DOI:** 10.1007/s00508-022-02082-3

**Published:** 2022-09-23

**Authors:** Gabriel Rinnerthaler, Christian Singer, Edgar Petru, Daniel Egle, Andreas Petzer, Ursula Pluschnig, Simon Peter Gampenrieder, Georg Pfeiler, Michael Gnant, Birgit Grünberger, Peter Krippl, Kathrin Strasser-Weippl, Christoph Suppan, Christine Brunner, Renate Pusch, Margit Sandholzer, Marija Balic, Rupert Bartsch

**Affiliations:** 1 Third Medical Department with Hematology and Medical Oncology, Hemostaseology, Rheumatology and Infectious Diseases, Oncologic Center, Paracelsus Medical University Salzburg, Müllner Hauptstr. 48, 5020 Salzburg, Austria; 2 Department of Gynecology and Gynecological Oncology, Medical University of Vienna, Währinger Gürtel 18–20, 1090 Vienna, Austria; 3 Department of Gynecology and Obstetrics, Division of Gynecology, Medical University of Graz, Auenbruggerplatz 14, 8036 Graz, Austria; 4 Department of Gynecology, Breast Cancer Center Tirol, Medical University of Innsbruck, Anichstr. 35, 6020 Innsbruck, Austria; 5 Barmherzige Schwestern, Elisabethinen, Department of Internal Medicine I for Hematology with Stem Cell Transplantation, Hemostaseology and Medical Oncology, Ordensklinikum Linz GmbH, Seilerstätte 4, 4010 Linz, Austria; 6 Department of Internal Medicine and Hematology and Internal Oncology, Klagenfurt Hospital, Feschnigstr. 11, 9020 Klagenfurt am Wörthersee, Austria; 7 Comprehensive Cancer Center, Medical University of Vienna, Währinger Gürtel 18–20, 1090 Vienna, Austria; 8 Department of Internal Medicine and Hematology and Internal Oncology, Landesklinikum Wiener Neustadt, Corvinusring 3–5, 2700 Wiener Neustadt, Austria; 9 Department of Internal Medicine (location Fürstenfeld), Landeskrankenhaus Feldbach-Fürstenfeld, Krankenhausgasse 1, 8280 Fürstenfeld, Austria; 10 First Medical Department, Center for Oncology and Hematology, Klinik Ottakring, Montleartstr. 36, 1160 Vienna, Austria; 11 Department of Internal Medicine, Division of Clinical Oncology, Medical University of Graz, Auenbruggerplatz 15, 8036 Graz, Austria; 12 Second Medical Department, Center for Oncology and Hematology, Landeskrankenhaus Feldkirch, Carinagasse 47, 6807 Feldkirch, Austria; 13 Department of Medicine I, Division of Oncology, Medical University of Vienna, Währinger Gürtel 18–20, 1090 Vienna, Austria

**Keywords:** Advanced breast cancer, Erbb2, Systemic therapy, Antibody-drug conjugates, Tyrosin kinase inhibitors

## Abstract

In the past 12 months a plethora of relevant novel data for the treatment of metastatic HER2 positive breast cancer were published. To bring this new evidence into a clinical perspective, a group of Austrian breast cancer specialists updated their previously published treatment algorithm for those patients. For this consensus paper a total of eight scenarios were developed in which treatment strategies appropriate for specific patient profiles were evaluated. Consensus was established by detailed discussions of each scenario and by reaching full consensus.

## Introduction

This consensus statement represents an update of previous work published as the *Updated Austrian treatment algorithm in HER2+ metastatic breast cancer* by Bartsch et al. [1]. The past 12 months have brought to light a plethora of relevant novel data [2–8] necessitating another update and an expansion of the recently established treatment algorithms for HER2-positive metastatic breast cancer (mBC).

Of particular interest are the results from the DESTINY-Breast03 trial. This prospective randomized phase III study compared trastuzumab-deruxtecan (T-DXd) directly with the former second-line standard of trastuzumab-emtansine (T-DM1) [2, 3]. In addition, an interim analysis from the DEBBRAH and TUXEDO‑1 phase II trials provided preliminary data on T‑DXd in patients with stable and progressing cerebral metastases [4, 8].

## Patients, material and methods

For this purpose, a group of leading Austrian breast cancer specialists have reconvened in January 2022 to detail eight scenarios for which treatment strategies appropriate for specific patient profiles were developed. These developments serve as an update to the four scenarios established in 2021 [1].

Data from the following sources serve as the basis for the clinical and scientific update of treatment recommendations: all studies included in the initial consensus statement [1], regulatory information on established and new compounds, scientific updates of the last 2 years from the following symposia/congresses: San Antonio Breast Cancer Symposium, the American Society of Clinical Oncology Annual Meetings, the European Society for Medical Oncology Annual Meetings, safety profiles and efficacy data of the respective compounds, current treatment recommendations for patients with HER2-positive mBC from various guidelines, and comprehensive clinical practice experiences of the respective experts, their teams and institutions.

Eight distinct scenarios were developed to evaluate treatment strategies appropriate for specific patient profiles including aspects of cerebral metastatic disease. Consensus was established through advisory board meetings, detailed discussions, and reiterations of clinical scenarios. Treatment recommendations for each specific scenario were finalized after reaching full consensus.

## Results

### New evidence from recent presentations and publications

DESTINY-Breast03 compared T‑DXd with T‑DM1 in patients with unresectable or metastatic HER2-positive breast cancer previously treated with trastuzumab and taxanes. This randomized, open-label, multicenter, phase III study [9] delivered the first randomized data set on T‑DXd which was approved in January 2021 based on phase II data from the DESTINY-Breast01 trial [10]. The trial demonstrated a clinically meaningful and statistically significant improvement in progression-free survival (PFS) of T‑DXd compared with T‑DM1 in patients with HER2-positive mBC (hazard ratio, HR 0.28; *P* = 7.8 × 10^22^) and an encouraging overall survival (OS) trend at the time of the first interim analysis (12-month OS rate was 94.1% for T‑DXd vs. 85.9% for T‑DM1). The safety profile was comparable between the two arms, showing similar rates of all grades and grade ≥ 3 drug-related treatment-emergent adverse events (TEAEs) and no grade 4 or 5 interstitial lung disease (ILD) or pneumonitis events in either arm [2].

In addition, preliminary results from the TUXEDO‑1 trial were presented at the ESMO 2021 meeting. This phase II trial evaluated the role of T‑DXD in patients with active de novo untreated brain metastases (BM) or BM progressing upon prior local therapy. In the first stage of a Simon-optimal two-stage design, six patients were included and five intracranial responses by RANO-BM criteria were observed [8]. A poster presented at SABCS 2021 on cohorts 1 and 3 from the Spanish DEBBRAH trial reported data on T‑DXd in patients with HER2-postitive mBC with stable BM after surgery, stereotactic radiosurgery (SRS) and/or whole brain radiation therapy (WBRT) (cohort 1) and in patients with HER2-positive mBC with progressing BM after surgery, SRS and/or WBRT (cohort 3) [4]. Preliminary data demonstrated efficacy with manageable toxicity in heavily pretreated patients with HER2-positive mBC with stable and progressing BM after local treatment. Given the small patient numbers in these trials, further investigation is required in larger cohorts to validate these findings and provide more complete evidence on the activity and safety of T‑DXd in this population [4, 8]. Still, preliminary results from TUXEDO‑1 and DEBBRAH provide the proof-of-principle of T‑DXd activity in active BM.

In addition, an analysis of 468 patients treated with T‑DXd from the French cohort temporary authorization for use program, which offered first real-world data was presented at ASCO 2021 [7]. Most of the patients had received trastuzumab, pertuzumab and T‑DM1 in previous treatment lines. T‑DXd showed a response rate of 56.7% which is comparable to the response rate reported in DESTINY-Breast01 of 62%. The safety profile of T‑DXd appeared manageable and no additional safety signals were observed in the cohort program. Of particular interest was the lack of high grade and/or fatal cases of ILD [7].

In addition, updated results from the HER2CLIMB study and tucatinib use in patient subpopulations were presented at ASCO 2021. The median OS benefit for patients in the tucatinib arm was reported to lie 5.5 months (24.7 months vs. 19.2 months, HR 0.73) over those in the placebo arm. All subgroups benefited from the addition of tucatinib [5].

An evaluation of the impact of tucatinib on health-related quality of life (HR-QoL) in HER2CLIMB demonstrated that HR-QoL was preserved for patients with HER2-positive mBC who were treated with tucatinib when added to trastuzumab and capecitabine. Additionally, HR-QoL was maintained longer with tucatinib therapy than with placebo among those with BM [11].

Ongoing trials are evaluating the efficacy of tucatinib in combination with trastuzumab and pertuzumab for HER2-postitive mBC (HER2CLIMB-05) [12] and in combination with T‑DM1 (HER2CLIMB-02) [13] and will provide insights into possible combination options for future therapeutic regimens.

A novel, not yet approved compound, trastuzumab duocarmazine (SYD985), demonstrated superiority over treatment of physician’s choice (TPC) in pretreated HER2-positive locally advanced or mBC in the pivotal phase III TULIP trial and may soon provide an additional treatment option in the armamentarium for this patient population [6]. SYD985, currently under the U.S. Food and Drug Administration review, demonstrated a statistically significant benefit in centrally reviewed, median PFS (7 months for SYD985 vs. 4.9 months for TPC; HR 0.64) and in investigator assessed PFS (6.9 months vs. 4.6 months; HR 0.60). The first OS analysis revealed a HR of 0.83. Adverse events of special interest included eye toxicity, which was higher in the SYD985 group (78.1%) compared to the TPC group (29.2%) and led to discontinuation of treatment in 22.9% of patients in the trastuzumab duocarmazine group. Interstitial lung disease (ILD)/pneumonitis was reported for 7.6% of patients (2.4% with grade 3 or higher) in the SYD985 arm [6]. More mature data are eagerly awaited and will provide clinicians with data on the potential future treatment positioning of SYD985.

### Development of the updated therapeutic algorithms and consensus creation for treatment scenarios

Each scenario and its respective treatment algorithm depicted in the applicable figure was developed by reaching full consensus between experts. All available evidence reported in the past 12 months from scientific literature, appropriate guidelines and recent symposia and conferences regarding HER2-positive mBC provided guidance in the establishment of these updated treatment recommendations. The recently published Austrian treatment recommendations for HER2-positive mBC [1] served as the core platform from which the adapted algorithms were developed.

The new scenarios are closely aligned with the recently published ESMO Clinical Practice Guidelines for the diagnosis, staging and treatment of patients with metastatic breast cancer [14]. The major extensions in this Austrian update relate to new data in specific treatment lines and the treatment algorithm of patients with BM.

### Scenario 1

**Fig. 1:**
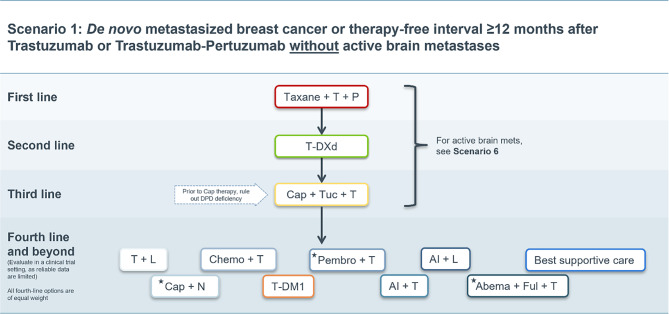
Treatment scenario 1. *T* trastuzumab, *P* pertuzumab, *T‑DXd* trastuzumab deruxtecan, *T‑DM1* trastuzumab emtansine, *Cap* capecitabine, *Tuc* tucatinib, *DPD* dihydropyrimidine dehydrogenase, *Chemo* chemotherapy, *L* lapatinib, *N* neratinib, *Pembro* pembrolizumab, *AI* aromatase inhibitor, *Abema* abemaciclib, *Ful* fulvestrant. ⋆These treatment options do not have market authorization in the EU

In patients with a treatment-free interval of ≥ 12 months after the end of adjuvant therapy or in de novo metastatic patients without active intracranial metastases, all approved substances are available according to current guidelines [15, 16]. The treatment algorithm for this scenario is depicted in Fig. 1.

Data generated by the CLEOPATRA study defined trastuzumab plus pertuzumab plus taxane-based chemotherapy (e.g., docetaxel) as the first-line therapy based on the improvement of PFS and OS. Dual HER2 inhibition plus chemotherapy demonstrated a clinically relevant OS advantage over trastuzumab plus docetaxel by a median of 16.3 months (HR 0.69; 95% confidence interval, CI; 0.58–0.82) [17]. For HER2-positive and hormone receptor (HR)-positive (luminal B/HER2-positive; triple positive) tumors, the addition of endocrine therapy to antibody maintenance therapy after completion of induction chemotherapy is recommended [15, 17].

In the second line setting, T‑DXd can be regarded as the new standard of care based on data generated from the DESTINY-Breast03 trial if no substantial contraindication for T‑DXd (e.g., severe pulmonary comorbidity) exists. Compared with the former second-line standard T‑DM1, T‑DXd demonstrated significant improvement in PFS (12-month PFS of T‑DXd 75.8 months; 95% CI; 69.8–80.7 months vs. T‑DM1 of 34.1 months 95% CI; 27.7–40.5 months) and a confirmed overall response rate (ORR) for T‑DXd of 79.7% vs. 34.2% for T‑DM1 (complete response, 16.1% vs. 8.7%), with a comparable toxicity profile [2].

In the third line, tucatinib in combination with trastuzumab and capecitabine may be used based on results from the HER2CLIMB trials [5, 11, 13, 18, 19].

According to European Medicines Agency recommendations, dihydropyrimidine dehydrogenase (DPD) deficiency testing should be performed prior to treatment with capecitabine, since patients with DPD deficiency are unable to metabolize capecitabine at a normal rate and may be at risk of life-threatening side effects [20]. The prevalence of DPD deficiency in Caucasians is 3–5% [20, 21].

Upon progression on tucatinib in combination with capecitabine and trastuzumab, no specific therapeutic recommendation according to the experts’ assessments and the current guidelines [15, 16, 22] can be given as data are scant with respect to efficacy in the fourth line and beyond. The individual needs and disease-specific factors of each patient must be considered to achieve the best possible outcome. Results from numerous studies suggest that the continuation of HER2 targeted therapy is beneficial [23–26]. A detailed data review for the compounds referenced in the fourth treatment line and beyond can be found in the previous edition of the Austrian HER2-postive mBC treatment recommendations [1].

### Scenario 2

**Fig. 2:**
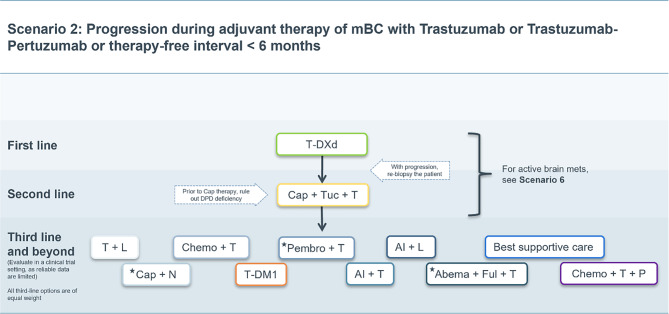
Treatment scenario 2. *T* trastuzumab, *P* pertuzumab, *T‑DXd* trastuzumab deruxtecan, *T‑DM1* trastuzumab emtansine, *Cap* capecitabine, *Tuc* tucatinib, *DPD* dihydropyrimidine dehydrogenase, *Chemo* chemotherapy, *L* lapatinib, *N* neratinib, *Pembro* pembrolizumab, *AI* aromatase inhibitor, *Abema* abemaciclib, *Ful* fulvestrant. ⋆These treatment options do not have market authorization in the EU

In patients with progression under adjuvant therapy with trastuzumab or trastuzumab plus pertuzumab or relapse within 6 months after the end of adjuvant therapy without active intracranial metastases, a rechallenge with trastuzumab, pertuzumab and chemotherapy in the first-line setting does not seem reasonable. Therefore, the second line standard of T‑DXd may be recommended as the first line therapy choice in this specific setting [14]. See Fig. 2 for therapeutic recommendation details. Consequently, all other therapeutic options described in scenario 1 above move up by one therapy line.

A rechallenge with trastuzumab plus pertuzumab in combination with chemotherapy in the 2nd, 3rd or 4th line may be considered. This seems particularly relevant for patients who have not yet received (neo)adjuvant pertuzumab and who have not favorably responded to neoadjuvant treatment.

The same considerations as in Scenario 1 apply to subsequent therapy lines.

### Scenario 3

**Fig. 3:**
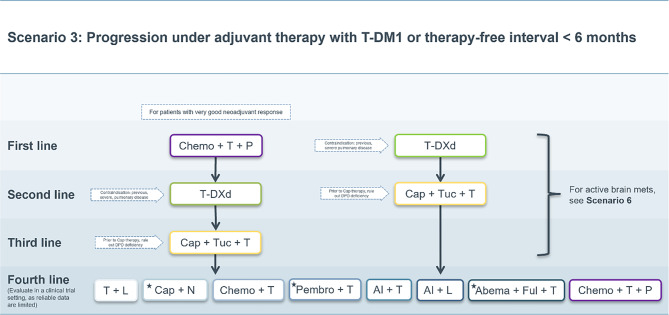
Treatment scenario 3. *T* trastuzumab, *P* pertuzumab, *T‑DXd* trastuzumab deruxtecan, *T‑DM1* trastuzumab emtansine, *Cap* capecitabine, *Tuc* tucatinib, *DPD* dihydropyrimidine dehydrogenase, *Chemo* chemotherapy, *L* lapatinib, *N* neratinib, *Pembro* pembrolizumab, *AI* aromatase inhibitor, *Abema* abemaciclib, *Ful* fulvestrant. ⋆These treatment options do not have market authorization in the EU

A special situation arises in case of systemic relapse during postneoadjuvant therapy with T‑DM1 or relapse within a treatment-free interval of 6 months or less and without intracranial metastases (Fig. 3).

If these patients have already received trastuzumab and pertuzumab in the neoadjuvant setting, the benefit of a rechallenge with trastuzumab, pertuzumab and chemotherapy is believed to be minimal. This may be assessed differently if a patient with initially large disease burden had not achieved pathological complete remission despite having a good treatment response. In general, in this setting, T‑DXd may also be recommended as first-line therapy. Subsequently, all other therapy options described in Scenario 1 (Fig. 1) above move up by one therapy line.

After second-line therapy (or third-line therapy if a rechallenge with trastuzumab plus pertuzumab plus chemotherapy was deemed appropriate), all therapeutic options described in the primary scenario subsequently move up by one treatment line.

### Scenario 4

**Fig. 4:**
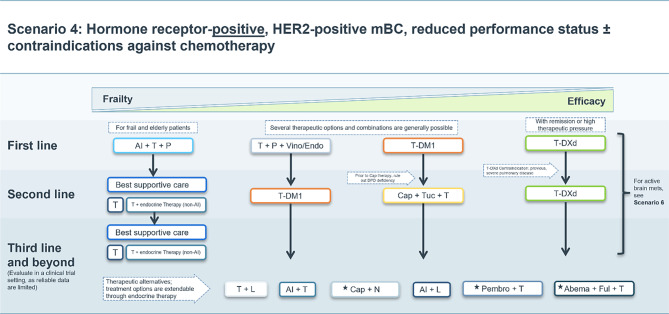
Treatment scenario 4. *T* trastuzumab, *P* pertuzumab, *AI* aromatase inhibitor, *Vino* vinorelbine, *Endo* endoxan, *T‑DXd* trastuzumab deruxtecan, *T‑DM1* trastuzumab emtansine, *Cap* capecitabine, *Tuc* tucatinib, *DPD* dihydropyrimidine dehydrogenase, *Chemo* chemotherapy, *L* lapatinib, *N* neratinib, *Pembro* pembrolizumab, *Abema* abemaciclib, *Ful* fulvestrant. ⋆These treatment options do not have market authorization in the EU

Patients with HR+, HER2-positive mBC, without active intracranial metastases, with or without contraindications against chemotherapy represent a specific subgroup. In this case, the currently valid therapeutic algorithm may be adapted accordingly as depicted in Fig. 4.

In unfit or old patients with HR-positive mBC, a combination of trastuzumab, pertuzumab and an aromatase inhibitor (AI) may be used as first-line therapy based on PERTAIN study data [27]. Further therapeutic lines for frail patients may represent single-agent endocrine therapy with a HER2-targeted treatment or best supportive care only. Of note, HER2-targeted therapy should only be omitted in cases of contraindications.

When the treatment regimen for older or frail HER2-positive mBC patients is expanded by adding metronomic oral cyclophosphamide to trastuzumab plus pertuzumab the median PFS was increased by 7 months compared with dual blockade therapy alone [28].

For less frail patients, data from the phase II VELVET trial have shown that first-line combination therapy with trastuzumab, pertuzumab, and vinorelbine is a safe alternative to standard (taxane-based) chemotherapy [29, 30]. Due to its acceptable toxicity profile T‑DM1 may be considered in this patient population for first line or subsequent treatment lines [31].

In fitter patients with high pressure for systemic treatment, a treatment algorithm like the one used for younger patients may be followed. This may include T‑DXd in the first line for patients with progression under (or shortly after) postneoadjuvant T‑DM1 therapy. Severe pulmonary comorbidities or related contraindications need to be considered prior to treatment initiation. In this population, the triple combination of tucatinib, trastuzumab and capecitabine may be considered a treatment option in subsequent lines. DPD testing should be performed prior to treatment with capecitabine.

Additional options may include trastuzumab plus lapatinib with or without an AI [31–35], capecitabine plus neratinib [36], pembrolizumab plus trastuzumab [37], an AI plus either trastuzumab or lapatinib, and abemaciclib plus fulvestrant and trastuzumab [38]; however, data supporting these treatment options in this population are very limited.

### Scenario 5

**Fig. 5:**
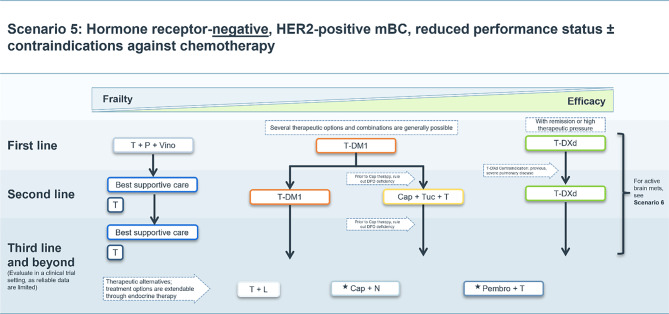
Treatment scenario 5. *T* trastuzumab, *P* pertuzumab, *AI* aromatase inhibitor, *Vino* vinorelbine, *T‑DXd* trastuzumab deruxtecan, *T‑DM1* trastuzumab emtansine, *Cap* capecitabine, *Tuc* tucatinib, *DPD* dihydropyrimidine dehydrogenase, *Chemo* chemotherapy, *L* lapatinib, *N* neratinib, *Pembro* pembrolizumab, *Abema* abemaciclib, *Ful* fulvestrant. ⋆These treatment options do not have market authorization in the EU

For older patients with HR-negative, HER2-positive mBC, without active intracranial metastases, depending on frailty and therapeutic pressure, trastuzumab, pertuzumab, and vinorelbine or oral cyclophosphamide, T‑DM1 or, depending on previous therapy, T‑DXd may be considered as a first line treatment option in selected cases and based on the clinician’s opinion (Fig. 5).

For very frail patients, trastuzumab in addition to best supportive care remains the best treatment option from the second line, while patients with a higher fitness level may benefit from treatments with T‑DM1, the triple combination of tucatinib, trastuzumab and capecitabine or T‑DXd.

In older or frail HER2-positive mBC patients metronomic oral cyclophosphamide added to trastuzumab plus pertuzumab increase the median PFS by 7 months compared with dual blockade therapy alone [28]. For less frail patients, data from the phase II VELVET trial have demonstrated a trastuzumab, pertuzumab, and vinorelbine combination therapy is a safe alternative to taxane-based chemotherapy [29, 30].

Alternatively, trastuzumab plus lapatinib [31–35], capecitabine plus neratinib [36], and pembrolizumab plus trastuzumab [37], may be explored as treatment options beyond the third line.

### Scenario 6

For patients with extracerebral progressive HER2-positive mBC with active BM and without an immediate indication for local therapy, the triple combination of tucatinib, trastuzumab and capecitabine can be considered the treatment of choice based on data from HER2CLIMB study [5, 13, 18, 19, 39]. Subsequently, T‑DXd may be given in the second line [2, 3].

In patients with a high extracranial tumor load and/or symptomatic or rapidly progressing extracranial disease, T‑DXd can be used as a first line treatment. In this case, the triple combination of tucatinib, trastuzumab and capecitabine will move to the subsequent treatment line.

**Fig. 6:**
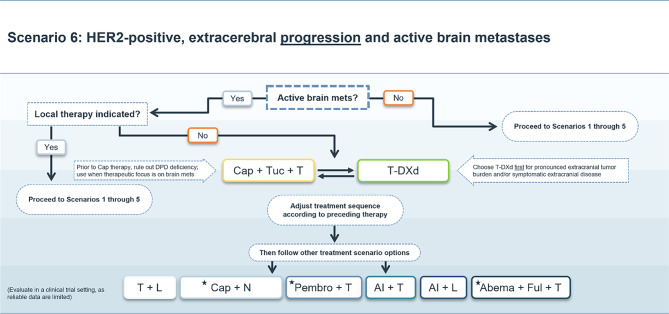
Treatment scenario 6. *Mets* metastases, *T* trastuzumab, *AI* aromatase inhibitor, *Vino* vinorelbine, *T‑DXd* trastuzumab deruxtecan, *Cap* capecitabine, *Tuc* tucatinib, *DPD* dihydropyrimidine dehydrogenase, *Chemo* chemotherapy, *L* lapatinib, *N* neratinib, *Pembro* pembrolizumab, *Abema* abemaciclib, *Ful* fulvestrant. ⋆These treatment options do not have market authorization in the EU

Subsequently, all other therapy options described in scenario 1 can be used, depending on previous treatments and the patient’s condition. (Fig. 6).

### Scenario 7

For patients with extracranial stable HER2-positive mBC with active BM in the absence of local treatment options other than WBRT, the treatment recommendation of Scenario 6 should be followed.

**Fig. 7:**
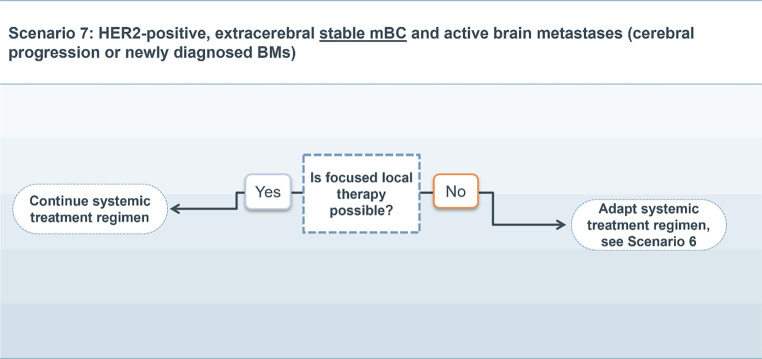
Treatment scenario 7

When local therapy other than WBRT is possible, the current systemic therapeutic regimen should be continued (Fig. 7).

### Scenario 8

For patients with HER2-positive mBC and exclusive intracranial metastases, and if local therapy is possible, the risks and benefits for observation versus systemic therapy need to be weighed against each other and adjusted to the needs of the individual patient.

**Fig. 8:**
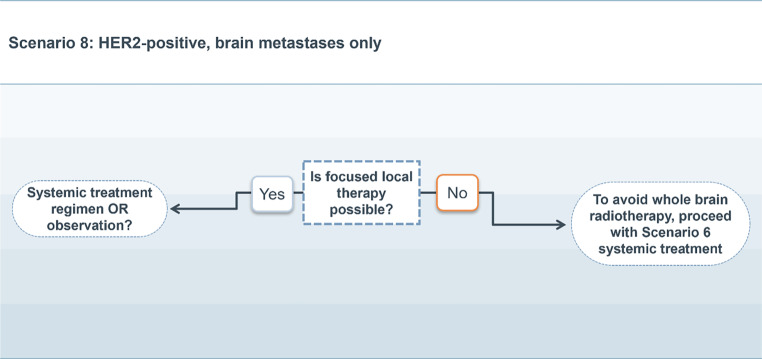
Treatment scenario 8

If local therapy is not indicated or possible, systemic therapy as described in scenario 6 should be followed to avoid or prolong the time to initiate WBRT. (Fig. 8).

## Discussion and outlook

Recently published data on novel agents like T‑DXd or tucatinib and the increasingly broad use of trastuzumab, pertuzumab, and T‑DM1 [9] in the adjuvant and neoadjuvant setting have led to increasing diversity in clinical settings, making the establishment of appropriate treatment algorithms more complex.

Due to the rapid progress in clinical research, a growing number of substances targeting HER2-positive disease are now available. Still, data partly remain insufficient to fully describe all scenarios and treatment lines needed in daily, routine clinical practice. In particular, the population of patients with HER2-positive mBC with BM warrant further research and require larger randomized trials to enable breast cancer specialists to make evidence-based decisions. In addition, the care for these patients requires a multidisciplinary approach with the inclusion of radiologists, radiation oncologists, neurosurgeons, medical oncologists, and gynecologists.

As numerous studies are currently ongoing and new evidence is eagerly anticipated for substances such as T‑DXd, tucatinib and SDY985, repeated further updates of treatment algorithms will be required.
